# Toward a true understanding of consciousness: the explanatory power behind the non-physicalist paradigm

**DOI:** 10.3389/fnhum.2026.1815678

**Published:** 2026-04-10

**Authors:** Joachim Keppler

**Affiliations:** Department of Consciousness Research, DIWISS Research Institute, Roth, Germany

**Keywords:** consciousness, explanatory power, metaphysical paradigm, non-physicalism, physicalism, predictive power, theory of consciousness

## Abstract

This article addresses the question of which metaphysical paradigm is most suitable for gaining a deeper understanding of our conscious inner life and bringing us closer to a powerful theory of consciousness (TOC). To answer this question, the key characteristics of a strong theory, namely, predictive and explanatory power, are used to evaluate various paradigms. The predictive power of a TOC relies primarily on how accurately it can state the conditions under which a physical system is capable of forming conscious states, whereas the explanatory power of a TOC reflects the degree to which the theory makes it intelligible why conscious states are formed under the stated conditions. It proves expedient for the evaluation to divide the paradigms into two classes: physicalism and non-physicalism. From the physicalist point of view, consciousness is reducible to the physical, while non-physicalism is predicated on the assumption that consciousness is fundamental and irreducible to physical properties. The analysis reveals that a TOC built on the physicalist paradigm has the potential to achieve high predictive power but fails to unfold explanatory power. It is demonstrated that the non-physicalist paradigm has clear advantages over physicalism when it comes to developing a powerful TOC. These findings make a strong case for initiating a paradigm shift that replaces the prevailing physicalist stance with a non-physicalist approach. Such a paradigm shift does not make the prominent neuroscientific theories obsolete. Rather, it places these theories in a broader context and entails a reinterpretation of the neurophysiological indicators of consciousness.

## 1 Introduction

One of the major challenges we face in the multidisciplinary field of cognitive science is to gain a deeper understanding of our conscious inner life, which is characterized by an enormous spectrum of *phenomenal qualities*, also referred to as *qualia*. Each specific conscious state we experience exhibits a unique phenomenal profile that determines what it is like for us to be in that specific state (Chalmers, 1995, 1996). It is this inner experiential world that requires a scientific explanation.

What does it mean to *understand* consciousness and what hurdles must be overcome before we can claim to have achieved a true understanding of consciousness? I take the position that a scientific understanding of consciousness is achieved as soon as we are able to (1) reconcile phenomenal qualities with our physical worldview, (2) correctly predict the phenomenal state of a given system provided we know its physical state, and (3) conclusively explain the physical conditions under which phenomenal states can occur. These three dimensions constitute the basis for the development of a theory of consciousness (TOC), with the first dimension being linked to the *paradigm* that underpins the theory. The paradigm, which sets the *metaphysical framework* for the interpretation of neurophysiological findings, is of great importance, as it can be the limiting factor for theory building or the door opener that points to new ways of thinking about conscious systems.

The scope of this paper is to set out the key characteristics of a powerful TOC (Section 2), to discuss various paradigms that can be considered for theory building, classified into physicalist and non-physicalist paradigms (Section 3), and to identify their potentials and limitations in developing a powerful TOC (Sections 4 and 5). The aim of the article is to show that the non-physicalist paradigm is more promising than the physicalist paradigm in guiding us to a conclusive theory that unveils the nature of consciousness, which makes a strong case for initiating a paradigm shift (Section 6).

## 2 Key characteristics of a powerful TOC

In accordance with the dimensions listed above, a strong theory stands out due to its predictive and explanatory power. High *predictive power* is achieved if a TOC delivers an accurate and reliable phenomenal *classification* of the states of a physical system, where the classification accuracy can be determined at various levels of granularity, from the differentiation between conscious and nonconscious states, to the distinction between different types of conscious states (modalities), right down to the prediction of the detailed phenomenal profile of conscious states. In other words, the predictive power of a theory relies primarily on how accurately it can state the conditions under which a physical system is capable of forming conscious states, and thus on how well it can define the dividing line between conscious and nonconscious states.

In contrast, the *explanatory power* of a TOC reflects the extent to which the theory is able to make us *understand* why the stated conditions are necessary (and sufficient) for the formation of conscious states, considering that the “goal of explanation is the creation of understanding” (Ylikoski and Kuorikoski, 2010). The crucial element in gaining understanding lies in “characterizing the mechanism by which the phenomenon is produced or realized” (Craver, 2008), with the mechanism providing *insight* into the cause-effect relationships underlying the phenomenon to be explained (Ylikoski and Kuorikoski, 2010). It is precisely this insight that is the hallmark of explanatory power, helping us to “decrease the degree to which we find the explanandum surprising” (Schupbach and Sprenger, 2011). In this sense, we can claim to have formulated a powerful TOC once we are able to present a mechanism that reveals the causal chain behind the formation of conscious states and makes it *intelligible* why conscious states are formed under the conditions deduced from this causal chain.

Uncovering the mechanism underlying conscious systems is regarded as the main goal of a TOC and widely recognized as one of the central ambitions of consciousness research (Fahrenfort and van Gaal, 2021; Francken et al., 2022; Seth and Bayne, 2022; Mudrik et al., 2025). Taking into account that the success in other fields of science, especially physics, is based on the concept of *universality* (Craver, 2008), it is reasonable to follow the guiding idea that all conscious systems are rooted in universal principles. Seen in this light, the explanatory power of a TOC is also closely linked to the universality of the proposed mechanism, because only by applying universal principles can we “extrapolate the theory to non-biological or non-brain-based systems” and gain “insights into the potential existence and characteristics of consciousness in other forms of life and artificial entities” (Kanai and Fujisawa, 2024).

## 3 Paradigms for the development of a TOC

Let us now turn to the metaphysical stances that come into question when attempting to construct a TOC. In preparation for the subsequent evaluation of the paradigms, it proves expedient to divide them into two main classes that are distinguished by their contrasting ontological premises.

I will subsume the first class of paradigms under the heading of *conventional physicalism*, which holds that consciousness is “*reducible* to or *grounded* in the physical” (Mørch, 2023). From the physicalist point of view, ultimate reality, as expressed in the basic blueprint of our universe, can be completely described by fundamental physical properties, while the phenomenal qualities characterizing conscious experiences can be traced back to physical states or processes and are therefore considered as *not fundamental*. Physicalism comprises various branches, the three most prominent ones being identity theory, according to which conscious processes are identical to specific brain processes (Place, 1956; Smart, 1959), emergentism, which takes conscious awareness to be a “dynamic emergent property” of complex activity patterns (Sperry, 1969, 1976), and computationalism (computational functionalism), which assumes that consciousness results from a special type of information processing (Putnam, 1967; Piccinini, 2004, 2009).

In summary, even though the various branches of physicalism differ in how they relate phenomenal qualities to physical properties, they agree that all phenomena arise from physical processes, rejecting the idea that phenomenal qualities are basic, irreducible ingredients of reality in the same way as space–time, energy, or charge are. In all branches of the physicalist family, phenomenal properties are assumed to be products of evolution that emerge once suitable physical conditions are in place.

These directions of thought are the starting point for a large part of the efforts aimed at naturalizing consciousness (Crick, 1994; Edelman, 2003) and constitute the foundation of a number of neuroscientific theories that we will encounter in Section 4. Given the causal closure of the physical world, the notion that conscious states are identical to neural activity patterns, arise from complex neural interactions, or are the outcome of neural computations seems to be for many scientists the most parsimonious approach to reconciling qualia with our physical worldview.

In contrast to conventional physicalism, the second class of paradigms, hereafter referred to as *non-physicalism*, is predicated on the assumption that qualia are *fundamental* and thus *irreducible* to physical properties (Mørch, 2023), meaning that phenomenal qualities are taken to be already inherent in the blueprint of the universe. This view is compatible with panpsychism, where two branches are to be distinguished. The first branch, termed micropsychism, rests on the idea that the elementary constituents of matter have intrinsic phenomenal qualities (Seager, 1995; Chalmers, 2013), while the second branch, known as cosmopsychism, is rooted in the concept of a cosmic level of consciousness that constitutes the ultimate ground of all conscious systems (Mathews, 2011; Shani, 2015; Goff, 2017; Nagasawa and Wager, 2016; Shani and Keppler, 2018; Keppler and Shani, 2020). Over the last few years, a scientifically sound variant of cosmopsychism has been formulated, positing that the universe is imbued with a ubiquitous field that exhibits extrinsic physical properties and intrinsic phenomenal qualities (Keppler and Shani, 2020; Keppler, 2021, 2024). In this approach, which also falls into the category of *dual-aspect monism* (Atmanspacher and Rickles, 2022), qualia constitute the intrinsic nature of the physical, meaning that consciousness is the realizer of physical structure and dynamics and occupies an “explanatory role compatible with physical causal closure” (Mørch, 2023). The rationale behind this approach is that for physical manifestations to exist, intrinsic phenomenal qualities are required, giving consciousness, rather than being merely epiphenomenal, an essential role that does not violate the causal closure of the physical world (Stoljar, 2001; Alter and Nagasawa, 2012; Chalmers, 2013). From this vantage point, dual-aspect monism is on a par with physicalism in terms of parsimony. Other lines of non-physicalism, such as idealism, are not dealt with here.

It is important to emphasize from the outset that the metaphysical premise of non-physicalism, which holds that qualia are ontologically basic, does not relieve a non-physicalist TOC of the explanatory burden. After all, it is not the case that all physical states are associated with qualia. The major challenge lies in explaining the conditions under which qualia are actualized in physical systems. These conditions must be consistent with empirical data, and a non-physicalist TOC possesses explanatory power only if it is able to provide a coherent explanation for the empirically supported conditions under which differentiated conscious states can occur.

## 4 Potential and limitations of the physicalist paradigm

The aim of the following analysis is to identify the potential, but also to expose the limitations of physicalism with regard to the development of a TOC. For this purpose, it suffices to examine a selection of contemporary neuroscientific theories, without performing an all-encompassing review of the theory landscape. A more extensive discussion can be found in Signorelli et al. (2021). Note that a neuroscientific and empirically grounded TOC does not entail adherence to the physicalist paradigm. The classification of the prominent neuroscientific theories under the heading of physicalism is due to the fact that all of these theories have their roots in the physicalist paradigm. The outlook in Section 6 will show that these theories can also be reinterpreted and viewed under the non-physicalist paradigm.

To begin with, we take a look at the neurophysiological features of consciousness. The empirical findings can be summarized in such a way that conscious states are associated with complex, large-scale, highly synchronized activity patterns (Rodriguez et al., 1999; Engel and Singer, 2001; Freeman, 2007; Melloni et al., 2007; Gaillard et al., 2009; Uhlhaas et al., 2009), each of which results from a phase transition and manifests itself in recurring neuronal avalanches (Beggs and Plenz, 2003; Plenz and Thiagarajan, 2007; Lombardi et al., 2014). All prominent neuroscientific theories draw on these empirical findings but disagree on which feature is essential. While some researchers assume the *synchronization* of various brain areas to be the core principle behind the formation of conscious states (Crick and Koch, 1990, 2003), others argue that *recurrent processing* is the key mechanism required to exceed the threshold of consciousness (Lamme and Roelfsema, 2000; Lamme, 2006). An alternative view holds that conscious states arise through the ignition of a distributed, large-scale assembly of interconnected neurons that form a *global workspace* (Dehaene et al., 1998; Dehaene and Naccache, 2001). In contrast, Integrated Information Theory (IIT) states that the dynamical complexity of neural interactions, reflected in an enormous range of transiently stable activity patterns and quantifiable in terms of *integrated information*, is the crucial feature of conscious processes (Tononi et al., 1994, 2016; Tononi, 2004, 2008; Oizumi et al., 2014).

Without making an assessment as to which of these theories is most consistent with empirical findings, it is plausible that a reliable differentiation between conscious and nonconscious states and thus a high degree of predictive power can be achieved on the basis of the criteria outlined above. But regardless of which criterion is best suited to draw the dividing line between conscious and nonconscious states, none of the presented mechanisms *necessitates* the occurrence of phenomenal qualities and makes it intelligible why consciousness emerges under the specified conditions. In other words, it remains *puzzling* why conscious states should be identified with or arise from integrated information, recurrent processing, the activation of a global neuronal workspace, or the synchronization of neural activity across brain areas. This explanatory gap lies at the core of the hard problem of consciousness (Levine, 1983; Chalmers, 1995, 1996; Block, 2009; Nagel, 2012), a problem that is considered intractable in principle starting from a physicalist paradigm, since there are no conceivable physical processes that would necessitate the occurrence of qualia (Strawson, 2006). This leads to the assessment that all prominent theories lack essential ingredients to satisfactorily explain the distinctive features between conscious and nonconscious processes and provide insight into the nature of consciousness (Lamme, 2018). It should be emphasized that this conclusion rests solely on the basic assumption that physicalism imposes on itself, namely that phenomenal qualities are not fundamental ingredients of ultimate reality, and results from what we accept as a satisfactory explanation. I argue that a satisfactory explanation is one that provides a deeper understanding of the mechanism behind the formation of conscious states.

We will conclude our analysis of physicalist approaches with the discussion of a more recent model that aspires to explain phenomenal consciousness in mechanistic biophysical terms. This is the Motivated Emotional Mind (MEM) model (Galus and Starzyk, 2021; Galus, 2024, 2025a, 2025b, 2025c), according to which consciousness arises from recurrent, top-down feedback loops, where higher cognitive areas project information back to lower sensory areas, reactivating them. It is postulated that this reactivation gives rise to a subjective experience. From this perspective, MEM views phenomenal consciousness as the brain’s secondary perception of its own internal states. Conscious percepts are assumed to be neuronal representations, called semblions, which are multi-level structures formed through repeated perception-action cycles.

The strength of MEM lies in specifying the conditions under which multi-faceted percepts can occur based on semblions, as the underlying structure formation process links multimodal sensory data with interoceptive states and results in the creation of a model of complex objects, observed scenes, and the perceived world, thus constituting an associative memory. However, the mechanism behind the formation of semblions does not explain why these percepts should be *conscious* percepts. This is because the core of the mechanism, which relies on recurrent, top-down feedback loops and the reactivation of lower sensory areas, does not make it comprehensible why these processes should give rise to qualia. In other words, the presented mechanism in combination with the physicalist ontology contains no element that would account for the emergence of phenomenal properties and thus make their occurrence less surprising.

In summary, a TOC built on the physicalist paradigm has the potential to achieve high predictive power, but it fails to unfold explanatory power. Consequently, physicalism is capable of describing the conditions for conscious states to arise, but it is incapable of giving deeper reasons for these conditions and arriving at a true understanding of consciousness.

## 5 Strengths of the non-physicalist paradigm

In what follows, it will be demonstrated that non-physicalism has clear advantages over physicalism when it comes to developing a powerful TOC. To this end, the strengths of the non-physicalist paradigm will be exemplified using an approach that has been continuously advanced in recent years (Keppler, 2013, 2016, 2018, 2020, 2021, 2024, 2025; Keppler and Shani, 2020).

The point of departure for this approach is the empirical finding that the complex neural activity patterns associated with conscious states (see Section 4) exhibit the key features of self-organized criticality (Linkenkaer-Hansen et al., 2001; Freeman, 2007; Kitzbichler et al., 2009; Chialvo, 2010; Tagliazucchi et al., 2012, 2016; Toker et al., 2022; Maschke et al., 2024). It turns out that quantum electrodynamics (QED), the fundamental theory of the electromagnetic interaction, provides the appropriate conceptual framework for getting to the bottom of self-organized criticality, with the crucial element of this framework being a ubiquitous ocean of energy, the electromagnetic zero-point field (ZPF), which occupies a prominent position in the edifice of modern physics. In its initial state, the ZPF is a stochastic field consisting of a spectrum of normal modes, none of which stands out from the other modes (la De Peña and Cetto, 1994, 1995). QED-based model calculations indicate that self-organized criticality arises from the resonant coupling of the ZPF to the glutamate pool of cortical microcolumns, involving macroscopic quantum effects that are essential for regulating the neuronal firing rate and establishing critical dynamics (Keppler, 2023, 2024, 2025). This implies that the ZPF plays a central role in understanding the activity patterns that are characteristic of consciousness and that *resonant brain-ZPF interaction is the fundamental mechanism underlying conscious processes* (Keppler, 2016, 2024, 2025). As a result of the resonant interaction, the internal structure of the ZPF is modified in such a way that the field modes dominating the coupling are amplified, suggesting that *the necessary condition for the formation of a specific conscious state is the amplification of specific ZPF modes* (Keppler, 2024, 2025).

These insights lead to well-defined, testable predictions and pave the way for a promising paradigm based on the hypothesis that *the ZPF is a dual-aspect field* composed of a spectrum of qualia intrinsic to the spectrum of normal modes (Keppler and Shani, 2020; Keppler, 2021, 2024). Starting from the stochastic initial state of the ZPF, which from the phenomenal perspective can be interpreted as an undifferentiated field of consciousness, the coupling mechanism causes the amplification of specific ZPF modes, which goes along with the activation of specific qualia inherent in the ZPF and can easily be construed as the actualization of a differentiated conscious state that emerges from the undifferentiated field of consciousness (Keppler, 2024). Note that due to the transition from a physicalist paradigm to a dual-aspect paradigm, the ZPF is regarded as a foundational dual-aspect entity and, hence, is to be interpreted as a psychophysical concept. This paradigm shift also assigns a dual meaning to other concepts, such as the resonance-induced amplification of specific ZPF modes, which can be interpreted as the activation of specific phenomenal qualities.

**Figure 1:**
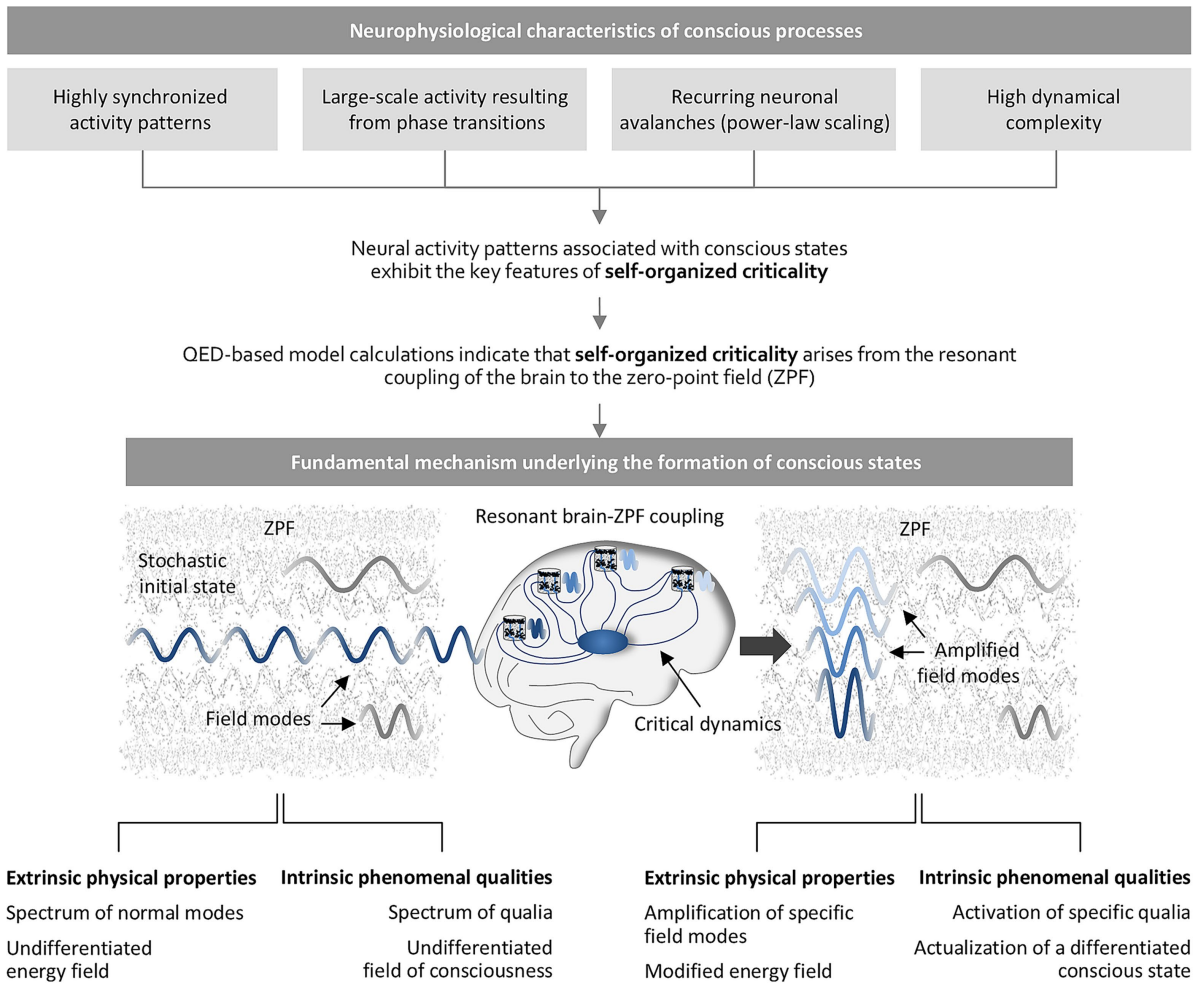
Explanatory power of the ZPF-based non-physicalist paradigm. Empirical evidence indicates that the neural activity patterns associated with conscious states display the key characteristics of self-organized criticality. QED-based model calculations reveal that self-organized criticality arises from the resonant interaction of the brain with the ZPF, suggesting that resonant brain-ZPF coupling is the fundamental mechanism underlying conscious processes. Assuming that the ZPF is a dual-aspect field with extrinsic physical properties and intrinsic phenomenal qualities, the causal chain behind the formation of conscious states becomes apparent from this mechanism. The crucial feature of the coupling mechanism lies in the amplification of specific ZPF modes, which goes along with the activation of specific qualia inherent in the ZPF, resulting in the actualization of a differentiated conscious state.

It is obvious that the dual-aspect paradigm has the potential to offer both predictive and explanatory power, as it reveals the causal chain behind the formation of differentiated conscious states (see Figure 1) and makes it *intelligible* why the formation of conscious states requires the necessary condition specified above (Keppler and Shani, 2020; Keppler, 2021, 2024). This condition means that conscious experiences are restricted to those systems that are capable of interacting resonantly with the intrinsically phenomenal ZPF, with the phenomenal profile of a particular conscious experience being determined by a particular set of amplified ZPF modes. Behind this interaction lies a *universal coupling mechanism* whose brain-specific realization is described in detail (Keppler, 2024, 2025), and which can be extrapolated straightforwardly to non-biological or non-brain-based systems (Keppler and Shani, 2020). It should also be emphasized that this mechanism opens a route to circumventing the combination problem of panpsychism (Seager, 1995; Chalmers, 2016; Shani and Keppler, 2018).

The aim of the proposed approach is to show that abandoning the physicalist paradigm and assuming that the ZPF is intrinsically phenomenal leads to a picture that is consistent with neurophysiological findings and at the same time offers explanatory power, with the explanatory power being rooted in a deeper insight into the fundamental mechanism underlying conscious processes. This insight makes the occurrence of conscious experiences less surprising, since the formation of differentiated phenomenal states can be traced back to the selective excitation of an intrinsically phenomenal field. Therefore, the approach makes it comprehensible why self-organized criticality is a hallmark of conscious processes, a degree of comprehensibility that cannot be achieved within the physicalist paradigm.

It should be pointed out that the presented coupling mechanism still needs to be empirically substantiated, meaning that the claims regarding the genuine predictive and explanatory power of the approach are subject to empirical testing. Specifically, two test scenarios can be considered: first, the detection of biophoton emission, which is a predicted concomitant effect of the resonant coupling of the ZPF to the glutamate pool of cortical microcolumns; second, the demonstration that the disruption of the coupling mechanism results in the absence of conscious states (Keppler, 2024, 2025). Clear evidence of glutamate-induced biophotonic activity, which is compatible with resonant glutamate-ZPF coupling, has been found in experiments conducted in the brains of rodents (Tang and Dai, 2014; Chai et al., 2018), supporting the predictive power of the proposed model. Future experiments should aim to test the predictions even more precisely using the energy spectra of the photon pulses. The central idea behind the second test scenario is to influence the structure of the ZPF in such a way that those ZPF modes that dominate the resonant coupling are selectively suppressed. Such a manipulation can be performed locally in a small brain region. By suppressing the dominant ZPF modes, resonant glutamate-ZPF coupling cannot be established, which is expected to cause the absence of conscious states that would normally be experienced. The required setup to test this prediction has been specified (Keppler, 2024), but the experiments have not yet been performed.

Pending full empirical corroboration, the presented mechanism is to be understood as a proposal intended to expose the strengths of a dual-aspect paradigm that links consciousness to the foundations of physics. The main benefits of this proposal lie in avoiding the hard problem that arises with any physicalist approach (Chalmers, 2013; Mørch, 2023), and in gaining a *deeper understanding of consciousness* beyond mere classification, as the conditions under which conscious states arise are made comprehensible.

## 6 Initiating a paradigm shift in consciousness research

Overall, there are strong arguments supporting a paradigm shift that replaces the prevailing physicalist stance with a non-physicalist, dual-aspect worldview, opening up new vistas for the development of a powerful TOC. Since in this worldview phenomenal qualities constitute the intrinsic nature of the physical, one can conceive of appropriate mechanisms and organizational principles through which specific conscious states can be actualized.

The non-physicalist paradigm proposed here entails a *reinterpretation* of the neurophysiological indicators of consciousness, suggesting that the complex neural activity patterns associated with conscious experiences should neither be equated with these experiences nor held responsible for the mysterious creation of these experiences, but be construed as hallmarks of the brain’s coupling to a foundational dual-aspect field. Such a paradigm shift does not make existing theories obsolete. Rather, the insights provided by the prominent theories are placed in a broader context. This can be elucidated using the example of IIT, postulating that the degree of consciousness of a system is fully determined by its dynamical complexity, which is linked to its cause-effect power and can be expressed in terms of integrated information, quantified by a measure called Φ (Tononi, 2008; Oizumi et al., 2014; Albantakis et al., 2023). In conjunction with recent research outcomes, according to which criticality is a crucial prerequisite for a large value of Φ (Arsiwalla and Verschure, 2016; Kim and Lee, 2019; Mediano et al., 2022) and self-organized criticality arises from the resonant interaction of a system with the ZPF (see Section 5), it can be concluded that a high degree of integrated information (Φ) is indicative of a system’s coupling to the ZPF. This speaks in favor of seeking the origin of consciousness in the ZPF and viewing consciousness as the intrinsic nature of this field, manifesting itself in cause-effect power—a notion that is consistent with the latest version of IIT (Albantakis et al., 2023). It is this new perspective that will open the door to a deeper understanding of our conscious inner life and lead us to a theory that unfolds explanatory strength.

## Data Availability

The original contributions presented in the study are included in the article/supplementary material, further inquiries can be directed to the corresponding author.
